# Compact Modeling of Allosteric Multisite Proteins: Application to a Cell Size Checkpoint

**DOI:** 10.1371/journal.pcbi.1003443

**Published:** 2014-02-06

**Authors:** Germán Enciso, Douglas R. Kellogg, Arturo Vargas

**Affiliations:** 1 Department of Mathematics, Department of Developmental and Cell Biology, University of California Irvine, Irvine, California, United States of America; 2 Department of Molecular, Cell and Developmental Biology, University of California Santa Cruz, Santa Cruz, California, United States of America; 3 Computational and Applied Mathematics Department, Rice University, Houston, Texas, United States of America; ETH Zurich, Switzerland

## Abstract

We explore a framework to model the dose response of allosteric multisite phosphorylation proteins using a single auxiliary variable. This reduction can closely replicate the steady state behavior of detailed multisite systems such as the Monod-Wyman-Changeux allosteric model or rule-based models. Optimal ultrasensitivity is obtained when the activation of an allosteric protein by its individual sites is concerted and redundant. The reduction makes this framework useful for modeling and analyzing biochemical systems in practical applications, where several multisite proteins may interact simultaneously. As an application we analyze a newly discovered checkpoint signaling pathway in budding yeast, which has been proposed to measure cell growth by monitoring signals generated at sites of plasma membrane growth. We show that the known components of this pathway can form a robust hysteretic switch. In particular, this system incorporates a signal proportional to bud growth or size, a mechanism to read the signal, and an all-or-none response triggered only when the signal reaches a threshold indicating that sufficient growth has occurred.

## Author Summary

A large number of proteins in the cell are modified post-translationally by phosphorylation at multiple specific locations. This can help bring about interesting dynamical behaviors such as bistability or all-or-none responses to stimuli. Such behaviors are in turn important for cellular decision-making, differentiation, or the regulation of cellular processes. In this paper we propose the use of a specific technique for modeling allosteric multisite proteins, which can be thought of as the reduced version of a more detailed set of reactions. After validating this technique computationally by comparison to more detailed systems, we apply it to a new model of a signal transduction pathway with several multisite proteins. The model is concerned with a mechanism in budding yeast that is thought to measure the extent of daughter bud growth and send a signal to initiate mitosis when sufficient growth has occurred. Using the given framework we derive an analytically tractable model that creates the desired all-or-none signal. Overall, we give quantitative support to a newly discovered biochemical pathway, and we show the utility of the new modeling framework in the context of a realistic biological problem.

## Introduction

Protein phosphorylation is a common form of post-translational modification frequently used in nature to alter protein activity, for instance by changing the electrostatic properties of the protein or its spatial structure. The phosphorylation of the same protein at multiple different aminoacid residues is also very common, and it is found in proteins such as p53 [1], Sic1 [2], EGFR [3], Wee1 [4], Ste5 [5] and many others [6].

The differences in the function of single-site vs. multisite phosphorylation are not completely understood. Many multisite proteins are involved in regulatory processes that can benefit from the presence of bistability, hysteresis, or limit cycles, which require sufficiently nonlinear interactions in addition to the right type of feedback [7], [8]. A reasonable hypothesis is that multisite phosphorylation can give rise to ultrasensitive dose responses, in a way that would not be possible in a comparable single-site system [9]–[13]. Many detailed mechanisms have also been proposed to explain the role of multisite systems in the emergence of bistability (for examples, see [14], [15]).

On the other hand, such detailed multisite mechanisms are normally not used as part of actual mathematical models of biochemical interactions. This is because explicitly modeling multiple sites usually involves the introduction of numerous variables, one or more for each phosphorylation state, and realistic models are often too complex already to justify this additional effort. Systems that attempt to model biochemical reactions explicitly often use the assumption that the protein has two states, one active and one inactive, with a simple reaction to transform one into the other, effectively assuming that the protein only has one site. Other models are more phenomenological in nature and include, for example, Hill function terms in the equation that are less clearly tied to the actual biochemical reactions [16], [17].

In this paper we describe a simple mechanistic approach for modeling multisite allosteric proteins. This approach, named modified fraction (MF) modeling, is capable of describing ultrasensitive dynamics without introducing a large number of additional variables. Under this framework one keeps track of the fraction of modified sites in the protein, and the concentration of active protein over time is estimated from this information. The protein is activated in a way that requires the phosphorylation of several but not all of the sites. The approximation becomes increasingly precise as the number of sites increases, with good estimates already for around four or more sites. In a sense, this mechanism can be considered a one-variable, quasi-steady state reduction of a model similar to the Monod-Wyman-Changeux allosteric system [18], although uses of MF modeling outside of MWC are also possible. The MF framework can also be extended to other types of multisite modification such as ligand binding, multisite transcription factor regulation, multisite methylation or acetylation, ubiquitination, etc [19], [20].

**Figure 1:**
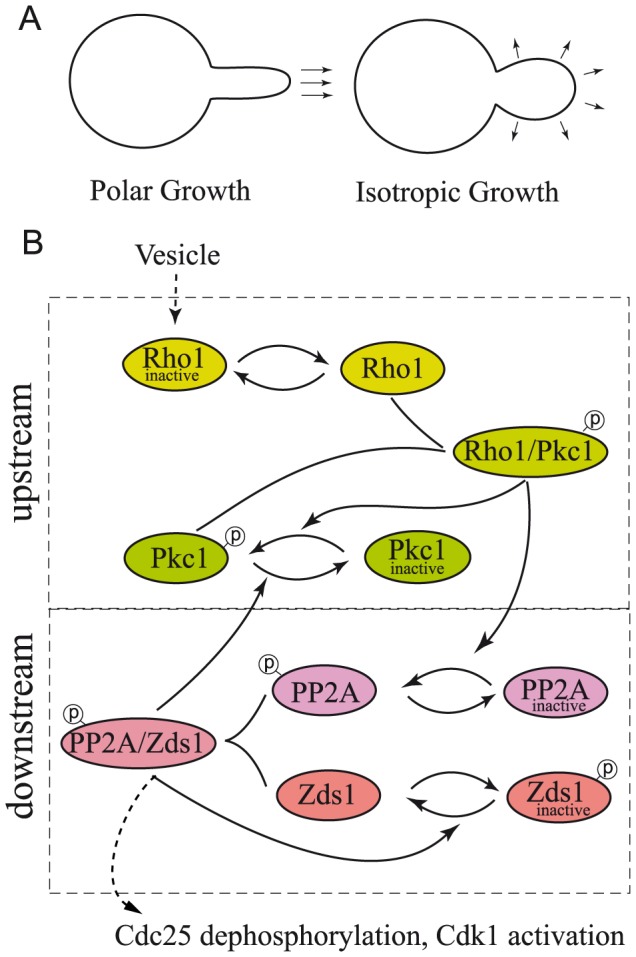
The Rho1 Network. A: A yeast bud grows first in a particular direction (polar growth) and eventually switches to growth in all directions (isotropic growth). B: The Rho1 signaling pathway starts with inactive Rho1 flowing into the bud attached to membrane vesicles. Rho1 is then activated and binds to Pkc1, forming an upstream system. The downstream system describes the activation of the PP2A/Zds1 dimer leading up to the modification of cell cycle regulatory protein Cdk1. Multiple intermediate feedback loops are shown to allow for robust hysteretic and switch-like behavior.

Perhaps the best way to test the versatility of a modeling tool such as the one proposed is to implement it in an actual biochemical system. In the current work we describe a detailed mechanistic model of a cell size checkpoint. Cell size checkpoints halt the cell cycle at specific points until sufficient cell growth has occurred [21], [22]. The mechanisms by which cell size checkpoints operate are poorly understood, and it is unclear whether they monitor actual cell size or parameters more closely related to the extent or rate of growth. In budding yeast, growth of a new cell is initiated when a daughter bud is formed on the surface of the cell [23]. The daughter bud initially grows in a polar manner, with all growth directed to the bud tip. Growth of the bud eventually switches to isotropic growth, in which the bud grows over its entire surface (see Figure 1A) [24]. The timing of the switch determines the duration of polar growth, which influences cell size and shape. It has been proposed in recent work by one of the authors that a cell size checkpoint controls the timing of this switch [25]. This checkpoint is the subject of our model. The variables are illustrated in Figure 1B and described in more detail below. See Tyson and Novak [26] for an accessible introduction to the systems-wide modeling of cell cycle checkpoints.

The MF approximation equation was first developed in [27], in the context of multisite systems with independent modification sites, with an emphasis on the estimation of the Hill exponents of sequential and nonsequential systems and on the comparison of their qualitative behavior. A major advance of the current paper, beyond the application to the cell cycle checkpoint, is to extend this work to cooperative and allosteric systems. Such systems are by definition non-independent, since the modification of one site accelerates the rate of modification of its neighbors. Cooperative systems are also more common and much better characterized than independent ones. The validation of the approximation in cooperative systems is ultimately based on a computational comparison of the MF reduction with detailed cooperative models having 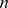 or 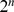 variables.

In the first two Results sections we carry out a description of the modified fraction method to model multisite systems, and we compare simulations of the reduced model with those of a detailed mechanistic model. In the remaining two Results sections we carry out a mathematical analysis of the proposed checkpoint signaling pathway. We hypothesize that this interaction pathway has the capacity to produce a bistable signal responsible for a sudden switch from polar to isotropic growth, once the bud has undergone sufficient polar growth. The model presents several desirable qualities for a checkpoint, in particular a clear downstream signal when a sufficient polar bud growth has occurred.

## Results

### The MF framework describes a compact multisite mechanism

We start by describing the assumptions on our model in the context of multisite phosphorylation (although it could also be applied to other irreversible covalent modifications as well as noncovalent ligand binding). Suppose that a protein substrate 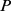 is phosphorylated by a kinase 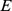 at 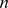 possible sites and dephosphorylated by a phosphatase 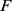. The system is assumed to be nonsequential, so that there are 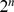 different phosphoforms of 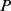, and the number of sites is thought to be relatively large e.g. 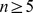. The system is cooperative in the sense that site phosphorylation accelerates the phosphorylation of neighboring sites. Since the number of sites is relatively large, the activation is thought to be cumulative and the effect of any individual site is assumed to be small. The sites are assumed to be equivalent to each other, in the sense that the rate of phosphorylation and dephosphorylation is similar across all sites and that no site has a stronger effect on substrate activation than other sites. The activation of the substrate may be due to binding to another molecule or body, such as the cell membrane. It could also be due to an internal structural change that allows the substrate to interact differently with other proteins. Thus the active protein concentration can be defined as the concentration of the protein bound to a particular molecule or in a particular molecular state.

Suppose that the phosphorylations lead to the concerted, redundant activation of the protein. That is, multiple phosphorylations are necessary for activation (concerted), and not all sites need to be phosphorylated for full activation (redundant). We define the activity function 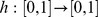 such that the fraction of active protein with 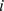 phosphorylated sites is given by 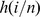.

There are many systems that likely fall within this general framework. For instance, Ste5 is a scaffold protein in budding yeast with 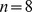 phosphorylation sites, which relays a pheromone response only when it is bound to the membrane [28], [29]. When phosphorylated by Cdk1, it tends to unbind from the membrane, shutting down its activity. The sites are predicted to lie on an unstructured region of the protein and appear to act by changing the protein's bulk electrostatic properties. In the paper [29], it was shown through site-directed mutagenesis that around five or more phosphorylations are necessary and sufficient for deactivation. Another recent example is the multisite phosphorylation of Cdc25 by Cdk1 in fission yeast, which was similarly studied in detail by mutating individual sites [30].

According to the modified fraction framework, we estimate the concentration of a particular protein state from the overall fraction of modified sites. For instance, if the protein has 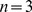 sites and the fraction of phosphorylated sites is 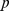, then the fraction of protein with only the first and last sites phosphorylated is roughly
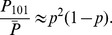
Here 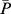 is the total protein concentration. This is not an equality since cooperative effects introduce correlations among the sites, i.e. the sites are not independent of each other, but it is an approximation assuming cooperative effects are sufficiently weak. Multiplying on both sides by 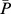 and adding over all possible phosphoforms with 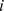 phosphorylations, the concentration 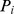 of proteins with exactly 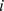 phosphorylated sites out of a total of 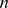 sites is
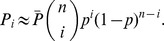
One can estimate the overall concentration 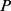 of *active* protein as
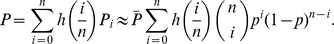
A key aspect of this formula is that if we denote the right hand side by 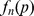, it can be shown that 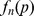 converges to 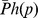 as 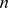 increases [31]. This gives the approximation
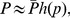
(1)which becomes increasingly precise for large 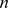. Notice that the different quantities in this formula can potentially be measured in the lab - the active protein concentration via an activity assay, the total protein concentration via Western blot, and the activity function 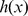 through site-directed mutagenesis.

A timescale decomposition argument can be made to use this approximation away from steady state. If 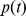 is the fraction of active sites over time, and the timescale of protein activation is much faster than the rate at which 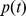 changes, then one can approximate 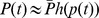 at any given time using the same formula. This produces a convenient method for modeling multisite systems under the given assumptions by keeping track of the variable 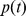, without creating 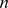, let alone 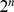 variables. On the other hand, if 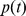, or any other process affecting protein activation, is at least as fast as protein activation itself, then nontrivial dynamics might take place such as limit cycle oscillations, and the approximation can introduce errors.

It is necessary to calculate the fraction of phosphorylation 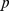 itself. Assuming linear rates of phosphorylation and dephosphorylation for 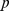, one obtains the system
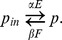



That is, 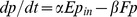, where 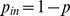 is the fraction of inactive sites. This is the default for the model, although any other rate equation for 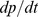 can be used, including Michaelis-Menten complex formation at the level of the individual sites.

As for the activity function 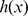, any sigmoidal function can be used, including functions measured directly by experiments. By default we assume the following form, which we will derive in the next section:
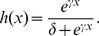



See also [5], [9], [27] for other uses and derivations of this formula in the literature. The function 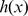 can actually be highly switch-like for large 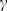, which illustrates how small increases in the kinase 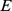 can result in large activity changes in the protein 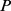, unlike linear rate models with only one site. Other forms for the activity function 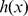 have effectively been considered by by Kapuy et al [32], and also by Wang et al [10].

**Figure 2:**
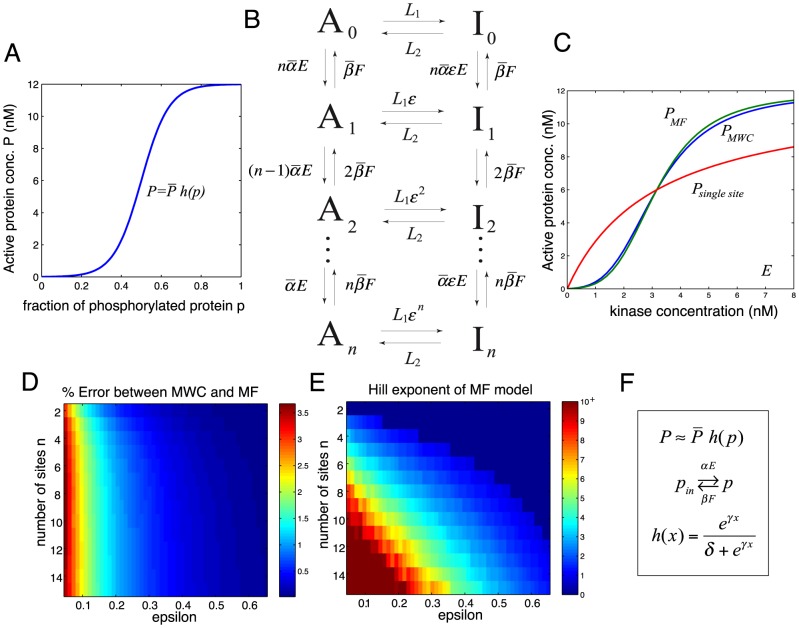
Comparison of MWC and MF models. A: The MF approximation formula is used to relate the fraction of phosphorylated protein 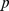 with the active protein concentration 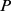. B: A detailed phosphorylation model structurally similar to the Monod-Wyman-Changeux model [18] is used to validate the MF approximation. C: The full MWC model is compared with the MF approximation at steady state as a function of kinase concentration 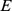. Also for comparison, a model in which it is simply assumed that the protein has a single site. Here 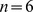, 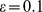, 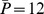 nM, 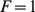 nM, 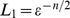, 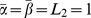 for the detailed model, and 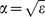, 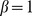, 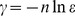, 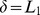 for the MF approximation. D: Comparison of the error between the MF and the MWC model, for different values of 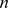 and 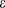 and the remaining parameters computed as above. E: Calculation of the Hill exponent of the MF model for different values of 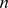, 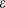. In each case 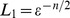, to allow for half maximal activation with 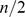 phosphorylations, and 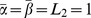. F: The default equations for the MF approximation. The active protein concentration 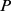 is a function of the fraction of active sites 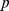 and the total protein 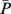. The values of 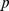 are calculated via a simple chemical reaction, and a form for the function 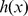 is suggested.

In Figure 2A we show the relationship between the fraction of phosphorylated sites 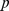 and the active protein concentration 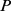 for a particular choice of the MF parameters 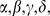 using the approximation formula 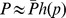 (and chosen to fit the detailed model described in the next section for 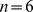). Notice that the activation is concerted and redundant, in that a minimal threshold of phosphorylation is required for activation, and activation is reached for less than full phosphorylation.

### The MF model reproduces the dynamics of detailed allosteric systems

A validation of the performance of this model for 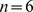 is now shown in Figure 2 in the context of a system similar to the classical and widely used Monod-Wyman-Changeux model of an allosteric multisite protein [18]. The original MWC model describes the binding of oxygen to the different sites of hemoglobin and the allosteric transitions of this protein between two different states. Rather than modeling oxygen binding, a protein with 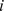 phosphorylations is assumed to change between an active conformation 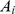 and an inactive conformation 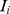. Each of these forms can also be phosphorylated or dephosphorylated at the rates given in the diagram in Figure 2B. Although the model is interpreted in a different way from MWC, from a mathematical point of view it is almost identical. The coefficient 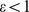 accounts for an assumption that the protein is phosphorylated at a faster rate when it is active than when it is inactive. The coefficients 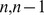 etc represent the fact that this is still a nonsequential model: for instance, 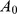 can be phosphorylated at 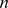 different sites, so the phosphorylation rate is multiplied by 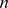. See the derivation of this model from more basic principles in Text S1, and a recent review on multisite systems by one of the authors [28].

Using this multisite model, we now derive parameters for a corresponding MF model. For instance, one can define the ‘average’ phosphorylation rate at a given phosphorylation site, regardless of whether the protein is active or inactive, 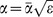, and the dephosphorylation rate 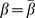. By way of derivation of the activity function 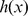, suppose that a protein with 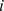 phosphorylations is switching between active and inactive form,
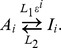
At steady state, we assume that this exchange is balanced and calculate 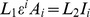. Then the fraction of active sites with 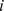 phosphorylations at steady state is
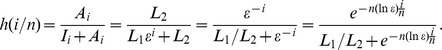
In other words 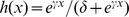, where 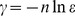 and 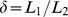. See Text S1.3 for more details. In particular, the ultrasensitive behavior of the function generally increases with the number of sites.

At any given time, the active protein concentration of the full model is defined as 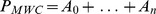. In Figure 2C we compare the full 12-variable MWC model for 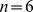 with the corresponding MF approximation. For every value of the input kinase concentration, the resulting concentration of active protein 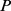 is plotted at steady state. Notice the close similarity between the two graphs, which is even more surprising since MF is essentially a one-variable model.

For comparison, we also plot the behavior of an overly simplified but all too commonly used model, in which the substrate is assumed to have a single phosphorylation site instead of 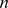 sites, and it is modeled according to the reaction
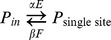
using linear reaction rates. Notice that the behavior of this single-site model in Figure 2C is very different from that of the MWC model, and that any switch-like behavior in the response is lost. This can have important consequences regarding the existence of multiple steady states, hysteresis, oscillations etc in the context of larger systems, which will be illustrated below. It is easy to show that 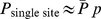, i.e. it corresponds to 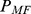 when 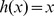. It should be noted that if the single site system is modeled using Michaelis-Menten reactions rather than linear rates, it could have strongly ultrasensitive behavior in the saturation regime via zero-order ultrasensitivity [33]; see the Discussion section for more details.

We carried out a calculation of the distance between 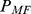 and 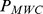 for many different combinations of the number of sites 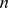 and the allosteric parameter 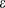. For every such set of parameters, the two graphs were plotted at steady state as a function of 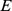, and the error 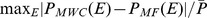 was calculated in Figure 2D. Notice that the approximation is within 1% precision for arbitrary 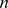 and 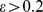. On the other hand, in order to obtain high ultrasensitivity it is required that 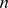 be relatively large and/or 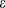 be small (Figure 2E). See also Figure S2, where additional parameter variations are explored over four orders of magnitude using the same type of graphs, with similar results.

It is worth comparing this methodology with the approach known in the literature as rule-based modeling, where a series of chemical reactions is defined using a streamlined algorithm, and high-powered computing is used to handle the resulting large number of variables; see e.g. BioNetGen [34]. The advantage of this method is that a large number of reactions can be defined and handled this way, including complex parameter optimizations. One disadvantage is that the combinatorial explosion resulting from combining reactions can sometimes exceed the computational power. Another is that the large number of equations makes any mathematical analysis difficult, if at all possible.

It is interesting that the MWC model can actually be described in terms of rule-based modeling. In Figure S1A we describe a series of chemical reactions, over all possible phosphoform states, and we show in Text S1 that this system is in fact equivalent to the MWC model. Thus MF can also be seen as the 1-variable reduction of a system with 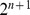 variables and a much larger number of reactions.

### Control theoretic analysis of the pathway reveals two robust switches

In this section, we will embed the MF system within increasingly complex systems of equations. We consistently use upper case for proteins and lower case for modified fractions of sites. However, we will first provide some technical experimental background regarding this specific pathway.

#### Background: The cell size checkpoint pathway

Any theory for cell size checkpoints should ideally account for several mechanistic features. First, cell size checkpoints should translate growth into a proportional checkpoint signal. Second, they should read the signal to detect when it reaches a threshold that indicates when sufficient growth has occurred. Finally, when the threshold is reached the checkpoint should trigger a switch-like cell cycle transition. All of these mechanisms should be robust and adaptable to function in cells of diverse size and shape, and under conditions of fluctuating growth rates.

It was recently hypothesized that the timing of the polar to isotropic growth transition described in the introduction is controlled by a mitotic cell size checkpoint. Mitotic cell size checkpoints are controlled by Wee1 and Cdc25 [25]. Wee1 is a protein kinase that delays entry into mitosis by phosphorylating and inhibiting Cdk1, while Cdc25 is a phosphatase that promotes entry into mitosis by dephosphorylating Cdk1. The budding yeast homologs of Wee1 and Cdc25 are known as Swe1 and Mih1; however, for clarity we will use their more commonly known names. In this model, a checkpoint signal originates at the site of polar membrane growth in the bud, and downstream components read the strength of the signal and trigger activation of Cdk1 when it reaches a threshold, using the pathway described in Figure 1B. A switch-like increase in Cdk1 during entry into mitosis is postulated based on evidence of this qualitative behavior of Cdk1 in other systems. Also, blocking polar membrane growth causes a Wee1-dependent arrest before mitosis, which indicates that entry into mitosis is linked to membrane growth [25]. The timing of the switch from polar to isotropic growth determines the duration of polar growth, which influences cell size and shape. In this sense, it is a cell size checkpoint.

Signaling is thought to be initiated by delivery of the GTPase Rho1 to the site of membrane growth. Rho1 is delivered by vesicles that are sent to the bud and fuse with the membrane to increase its size [35]. Therefore the inflow of Rho1 is proportional with the rate of polar membrane growth. Rho1 on vesicles is inactive and it undergoes activation when vesicles fuse at the site of growth [35]. Active Rho1 binds protein kinase C (Pkc1), a member of the protein kinase C family, and induces it to undergo autophosphorylation [36]. Importantly, phosphorylation of Pkc1 is dependent upon and appears to be proportional to membrane growth, which suggests that Rho1 relays proportional checkpoint signals regarding the extent of membrane growth [25]. This scenario describes the upstream component of the pathway in Figure 1B.

Regarding the downstream component in Figure 1B, it is thought that hyperphosphorylation of Cdc25 by a poorly understood kinase inhibits its activity [37], [38]. Thus, a key event necessary for initiation of early mitotic events is dephosphorylation of Cdc25. This dephosphorylation is carried out by the phosphatase PP2A (more precisely, the heterotrimeric complex PP2A^Cdc55^ that includes the Cdc55 regulatory subunit [25]). PP2A is in turn activated by the upstream Pkc1 protein. Activation of PP2A is also dependent upon an accessory protein named Zds1 [25], [38] (Zds1 has a redundant paralog named Zds2, which will be identified with Zds1 in the model). Zds1 forms a tight stoichiometric complex with PP2A [25]. It also binds to Pkc1, although that bond is not included explicitly in the model. PP2A is also believed to dephosphorylate Pkc1. Thus, inactivation of PP2A causes hyperphosphorylation of Pkc1 [25]. Although the functions of Pkc1 phosphorylation are not yet fully known, this observation suggests that PP2A restrains activation of Pkc1 by checkpoint signals. Similarly, PP2A also dephosphorylates associated Zds1, which suggests that activation of PP2A by Pkc1 leads to dephosphorylation of Zds1 and activation of targeted dephosphorylation of Cdc25 [38]. In this way PP2A is at the heart of checkpoint signaling: it opposes phosphorylation of both Zds1 and Pkc1, and it is responsible for the critical step of dephosphorylating Cdc25. There is also good evidence that PP2A regulates Wee1, although it is less clear whether this function of PP2A is controlled by signals from Pkc1 [4], [38].

#### A bistable switch between Zds1 and PP2A

We can now analyze a model involving two different multisite proteins, Zds1 and PP2A, each of them described according to the MF framework. This forms the downstream component of a larger model for the signal transduction of Rho1, and it is described inside the lower dashed rectangle in Figure 1B.

The activity of each of the two proteins can be modified through phosphorylation and dephosphorylation, and both proteins bind together to form a dimer. Only the dimer configuration of these molecules is active as an enzyme, and only to the extent that the Zds1 and PP2A components have been modified appropriately through phosphorylation or dephosphorylation. Moreover, as shown in the figure and justified by experimental data, the active dimer is itself involved in the modification of the Zds1 protein.

Denote by 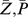 the total monomer concentration of Zds1 and PP2A respectively, regardless of their phosphorylation state, 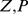 their active concentration, 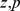 the modified fraction of Zds1 and PP2A sites, and 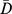 the total Zds1/PP2A dimer concentration. Call 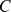 the active Rho1/Pkc1 dimer concentration, which can be treated as a constant input to the system for now. The dimerization reaction can be written as
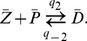



Now, following the argument in the previous section and given that both Zds1 and PP2A have been found to have 5 to 10 phosphorylation sites each, we keep track of 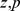 using the equations
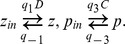



Since Zds1 is activated through dephosphorylation, here 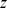 represents the fraction of *dephosphorylated* Zds1 sites, while 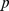 represents the fraction of phosphorylated PP2A sites. Using the MF equation one can then estimate
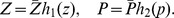



**Figure 3:**
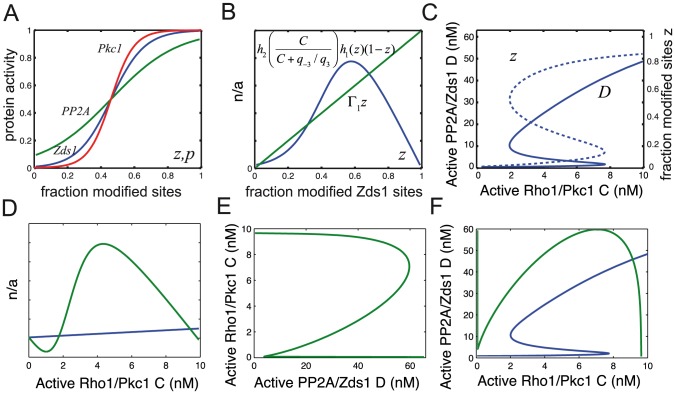
Control theoretic model analysis. The overall model is analyzed by decomposing it into upstream and downstream levels. A: The functions 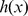 used to describe the activation of Zds1, PP2A and Pkc1. B: The solutions of the downstream Zds1 - PP2A system (for fixed 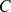) correspond to the intersections of the two graphs; see equation (2). C: Bifurcation graph for the downstream Zds1 - PP2A system. D: the solutions of the upstream Rho1/Pkc1 system (for fixed 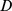) correspond to the intersections of these graphs; see equation (3). E: Bifurcation graph for the upstream Rho1 - Pkc1 system. F: Both bifurcation graphs superimposed - the steady states of the full model correspond to the intersection of these two graphs.

See Figure 3A for a graph of these two functions. The level of ultrasensitivity of each curve is dependent on the estimated number of sites in each protein (see the Methods section), but notice that the graphs are roughly consistent with the measurements done for example in the Ste5 protein. A simple mathematical analysis of this 5-dimensional model in Text S1.1 shows that the solutions of the system converge towards the steady states, and that the steady state equations can actually be reduced to a single equation for 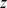,
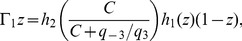
(2)where 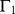 can be calculated from the system parameters. For a given value of 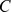 this equation can have one or possibly three solutions, depending on the parameter values. Plotting the left and right hand sides separately can be helpful (Figure 3B). As 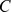 increases, the function 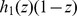 is rescaled vertically, which can control the number of solutions and create a hysteretic response on the variable 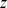.

Given a solution for 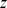 in this equation one can solve for the concentration of 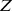, 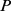 and every other protein. In particular one can calculate the concentration of the active form of the Zds1/PP2A dimer which we call 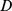, and which constitutes the natural output of this system. For specific values of the parameters in the model, a bifurcation graph of the output 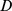 as a function of the input 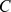 can be found in Figure 3C (solid line).

One can interpret this graph in the following way. There is a positive feedback loop in the system consisting of Zds1 promoting its own activation via dimerization with PP2A. This positive feedback allows the possibility of two stable steady states in the system. When 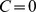, there can be binding between Zds1 and PP2A but since PP2A is inactive, most of the PP2A/Zds1 dimer is also inactive. For high values of 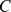, PP2A is forced to become active, however the PP2A/Zds1 dimer may still have two steady states or only one, depending on the parameters of the system.

#### A bistable switch between Rho1 and Pkc1

In a similar way as it was carried out for the interaction between Zds1 and PP2A, one can study the activation of Rho1 and its binding with Pkc1. This upstream system includes all the variables and interactions that are not in the Zds1/PP2A module. Once again following the biological evidence and the diagram in Figure 1B, we define the biochemical reactions
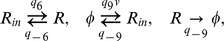
where 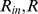 denote inactive and active Rho1 in monomer form respectively and 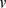 the rate of vesicle fusion to the membrane. That is, the protein Rho1 is activated at a linear rate, the vesicle flow increases Rho1 concentration in inactive form, and both active and inactive Rho1 are degraded. The protein Pkc1 is modeled using a MF mechanism and denoted by the variables 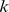, 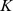:
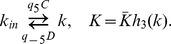



For the study of this subsystem, 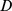 will be considered a fixed input parameter; it constitutes the feedback from the downstream Zds1/PP2A system. Calling 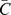 the total Pkc1/PP2A dimer regardless of activity,
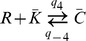



Notice that Rho1 needs to be active to bind with Pkc1. However active Rho1 binds with Pkc1 regardless of the activity or inactivity of the latter. Pkc1 does need to be active in order for the dimer to become active as a kinase, so that 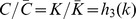, or
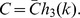



Once again, one can carry out a complete mathematical analysis of this multi-dimensional system at steady state and essentially reduce its solutions to one equation for 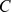. The resulting equation is
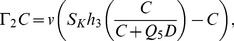
(3)where 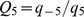, 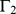 can be calculated from the parameters and constants of the system (other than 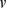, 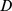), and 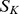 is constant. See Text S1.2 for a full analysis.

Figure 3D illustrates the solutions of the above equation in the general case, plotting right and left hand sides separately. For fixed 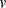 and 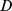 values, 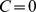 is always a solution, however there may also be solutions 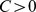. For increasing values of 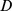, the values decrease on the right hand side of the equation, and the steady state 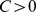 eventually disappears. Figure 3E illustrates a typical bifurcation graph of the steady states of 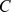 using the input parameter 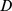 and fixed 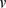. In the case 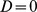 there is actually only one solution for 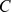, since equation (3) becomes linear.

The bifurcation graph can be interpreted as follows. For all but small values of the PP2A/Zds1 dimer 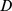, there is the steady state with 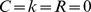, in which the pathway is inactive since the lack of active Rho1 inhibits the activation of Pkc1. For middle concentrations of 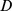, the system may be bistable, again due to the positive feedback from Rho1/Pkc1 to the activation of Pkc1. For high concentrations of 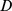, Pkc1 is almost fully inactivated by 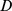 and the pathway is shut down.

### The Rho1 pathway can implement a cell size checkpoint

In order to find the steady states of both subsystems together, recall that each one can be reduced to a single equation, so that the steady states correspond to the joint solution of the two equations. For fixed 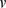, the solutions of the full model form the intersection of the graphs for the equations (2), (3). This is illustrated in Figure 3F, where the graphs in Figure 3C and Figure 3E are superimposed on the same plane. From a control perspective, the upstream and downstream systems have each an input and an ouptut, and they feed back into each other (see the two dotted boxes in Figure 1B). The active Zds1/PP2A dimer 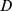 also acts as the overall output of the system, since it triggers the downstream response to cell cycle regulatory proteins.

Although it is natural that an increase in the Rho1 flow can eventually trigger the activation of the pathway, the main focus here is not in the flow but in the overall Rho1 concentration at the bud tip. Given that Rho1 has a rate of growth proportional to 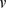 and a linear rate of degradation, at steady state one can show that 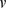 and 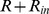 are proportional, 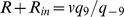. This follows from adding the ODE rate equations at steady state, 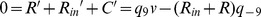. In this way one can use the total Rho1 concentration 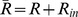 as a bifurcation parameter at steady state even though it is simultaneously a variable in the system. Alternatively, since enzymatic reactions and dimer formation are fast processes compared with Rho1 flux and Rho1 degradation, one can let 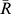 be the slow variable in the system and carry out a timescale decomposition analysis using 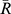 as a constant [39].

**Figure 4:**
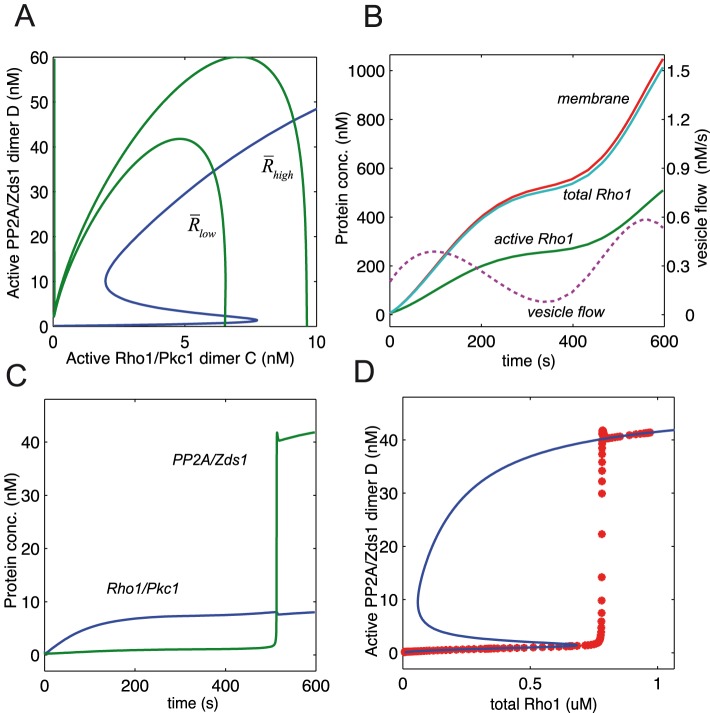
Checkpoint analysis. A: Qualitative analysis of the steady states of the system for different values of the total Rho1 concentration 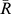, as a result of changing the value of the fixed flux rate 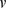. When 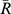 reaches a sufficiently large value there is a single positive steady state and it has a large 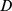 concentration. B,C: Response of the checkpoint pathway to a variable vesicle flow input (dashed line). For simplicity the membrane is modeled using the equation 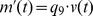. D: The steady state values of the output 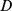 as a function of 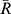 (solid line), and plot of the timecourse in 4C over time (stars).

Let's look at how the system has a hysteretic response for increasing values of the flow signal 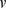 and the corresponding total Rho1 concentration at steady state. An increase in these values has the effect of raising the graph associated with the upstream system, as shown in Figure 4A. For smaller values of 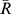, the intersection of both graphs includes three positive steady states (notice the two graphs don't quite intersect at the origin). But when 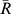 increases over a certain threshold, the intersection of the two graphs contains a single positive steady state, with a large value of 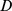. This can cause an abrupt change in the qualitative behavior of the system, triggering a sudden increase in the Zds1/PP2A output. Once this change has taken place, the concentration of the output stays high even if the input decreases.

Figure 4B shows a sample timecourse of the system for a time-variable vesicle flow (dotted line). The total Rho1 concentration increases over time with the inflow of vesicles. At a certain timepoint the active Rho1 concentration abruptly increases, due to the switch at the Pkc1/Rho1 upstream level. An increase in Rho1/Pkc1 concentration some time before this can be seen in Figure 4C. At a later time the switch between Zds1 and PP2A is also triggered, leading to a sudden increase in PP2A/Zds1 concentration. Even under variable flow, the total Rho1 concentration roughly corresponds to the membrane accumulated at the bud, except for a certain amount of variability due to Rho1 degradation. Lowering the Rho1 degradation rate can decrease this difference. Notice that the vesicle flow oscillations do not correspond to cell division, but to oscillations in the rate of growth, for instance due to varying food availability.

In Figure 4D we plot the output signal PP2A/Zds1 as a function of total Rho1 at steady state and overlay the solution of the timecourse simulation (red stars). This graph also illustrates the hysteretic behavior of the system, in that once a critical threshold of Rho1 concentration is reached, the output signal is dramatically increased. This change would constitute a clear signal that the bud has reached a large enough size for crossing the polar/isotropic growth checkpoint.

Since both the downstream (PP2A, Zds1) switch and the upstream (Rho1,Pkc1) switch are driven by positive feedback loops, it is valid to ask which of the two loops is more relevant for the overall system behavior. We argue that it is the downstream loop that is more essential, using the bifurcation analysis in Figure 3 and Figure 4A. If the upstream system is not bistable but has a single steady state for every input 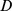, then the graph in Figure 3E is replaced by a single-valued decreasing function. Nevertheless this (green) line can still have one or three intersections with the (blue) downstream dose response in Figure 4A, indicating hysteresis for the overall system. On the other hand if the downstream system is not bistable, then the blue curve in Figure 4A is replaced by a single-valued, increasing function, which would be unlikely to have three intersection points with the (green) upstream dose response. Thus the downstream switch is essential, while the upstream switch is not.

Notice that this system contains the standard elements of a signal transduction pathway, including an initiating signal (vesicles), a sensor (Rho1), a series of transducers (Pkc1, PP2A, etc), and an effector (active Zds1/PP2A). Total Rho1 is a proxy for the membrane concentration, even if bud growth slows for a period of time, and the cascade of reactions allows the signal to be transduced from the membrane to Zds1/PP2A and ultimately the cyclin dependent kinase. To ensure the high fidelity of the signal transmission [40], the downstream signal is sent abruptly after total Rho1 concentration reaches a particular size. Notice that longer periods of inactivity can potentially reduce the Rho1 concentration significantly – one possible prediction is that after such a period the bud grows longer than expected.

## Discussion

In this paper we have introduced a simple and compact framework to describe the dynamics of allosteric multisite phosphorylation systems, and we have applied this tool to a new molecular model of a size checkpoint in budding yeast. Multisite phosphorylation modeling can be problematic because ignoring the multiple sites can have significant effects in the dynamics, while introducing many auxiliary phosphoform variables can be cumbersome in more realistic models. The modified fraction approach is intuitive and flexible (model the sites instead of the protein), and it only introduces one additional variable per protein.

The components of the MF model, namely the function 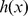 and the rates of phosphorylation and dephosphorylation of individual sites, can potentially be subject to direct experimental measurement, unlike the use of more abstract Hill function terms. This can allow to carry out ‘raw-data modeling’ e.g. to use an experimentally measured activity function 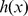 directly in the model rather than using it to derive parameters. This methodology is also useful out of equilibrium when the timescale of phosphorylation is sufficiently fast compared with other timescales in the system.

There are several reasons why the MF method might be particularly suitable for modeling many multisite phosphorylation systems. Nonsequential phosphorylation is likely more common in nature than the more often modeled sequential systems, since enforcing sequential phosphorylations would require an additional mechanistic effort. Bioinformatic data suggests that most phosphorylation sites in multisite proteins are located in unstructured and unconserved protein regions [6], suggesting that often it is the collective effect that matters rather than the individual sites. There is also experimental evidence in yeast signal transduction that certain proteins, such as Ste5, are activated in a concerted and redundant manner, although this type of information is still unknown for most proteins. Notice that the approximation formula would still hold if the protein activation is not concerted or redundant. In that case the formula will just approximate a dose response that may not be ultrasensitive.

One of the best known mechanisms for ultrasensitive dose responses is zero-order ultrasensitivity, as suggested by Goldbeter and Koshland [33], [41]. Its main assumption is that substrate concentration needs to be in the saturation regime i.e. large compared to the 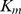 value of the enzymes. The MF method does not pose any constraint on 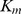, in fact the linear regime we used can be found when substrate concentrations are small compared to 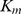 values. Moreover, MF also applies when the enzymatic reactions involve complex formation, by writing a Michaelis-Menten equation for 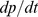. Therefore zero-order ultrasensitivity can be used in synergy with MF in the saturation regime, and MF can be used regardless of 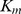 value. A zero-order dose response could likely replicate the behavior of the MWC model as shown in Figure 2C, however it could not be considered a short hand notation for MWC since the two mechanisms are fundamentally different.

In the case of the checkpoint pathway, the active proteins Pkc1 and PP2A have been found to have an approximate 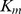 of 0.5 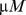 [42] and 1.2 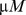 [43], respectively, for specific targets. The overall concentrations of their substrates in the cell are much lower - however these proteins tend to localize at the bud, so that the resulting local concentrations are unknown and it is unclear whether a zero-order approach would apply. A recent paper by Martins and Swain [44] points out that zero-order ultrasensitivity often results from low enzyme to substrate ratios, and localized proteins that act as enzymes and substrates for each other would likely not satisfy such ratios. That paper proposes instead a mechanism involving an allosteric model analogous to MWC, using enzyme sequestration to obtain ultrasensitivity. The paper by Kapuy et al [32] also proposes a mechanism for bistability through ultrasensitive effects, and this mechanism is applied to a detailed model of the budding yeast G1-S transition in Barik et al [45]. Other mechanisms for ultrasensitivity involve competition among substrates for the same enzyme [46] and protein localization [12], among others [28], [47].

More generally, in cell regulatory networks there is a need to implement nontrivial dynamics such as bistable switches and hysteresis, which requires some form of nonlinear response in addition to the right feedback interconnections. It has been observed that several regulatory proteins have multiple phosphorylation sites, and there are many open questions regarding their intended function. Together with the onerous nature of modeling several multisite proteins using sequential networks and multiple variables each, it can be seen why a one-variable reduction such as MF can allow for much-needed simplicity.

The actual mechanisms regulating the interactions between cell size and cell division remain largely unanswered in many cases. This has left few alternative options apart from somewhat heuristic approaches in otherwise very detailed models, see e.g. [48]. The present model is an attempt, based on recent experiments, to construct a detailed mechanistic model in the context of the polar to isotropic bud transition in yeast. Notice that if the proteins PP2A, Pkc1, Zds1 had only one site each, then 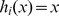 according to the argument in the first Results section, and the downstream and upstream models could never be bistable (see equations (2) and (3)). The multiple sites are providing the underlying nonlinearity so that the models can have interesting dynamical behaviors. This is consistent with the work by Yang et al [49], which reached the same conclusion through randomized parameter searches in multisite cell cycle models. Also, the equations (2), (3), which represent the steady states of the downstream and upstream systems, have the same qualitative behavior for a wide range of parameters. In this sense one can say that the switch-like nature of the checkpoint is robust to many parameter changes, provided that a few key qualities are satisfied. The bistability in each subsystem is due in part to positive feedback loops in each subsystem, one between Pkc1 and the Rho1/Pkc1 dimer, and another between Zds1 and the PP2A/Zds1 dimer. Notice that while Rho1/Pkc1 activates PP2A, the downstream PP2A/Zds1 inactivates Pkc1, forming a negative feedback loop. This feedback could serve to reduce the activity of the pathway before a sufficient Rho1 signal has accumulated. Notice that the switch-like activation of Cdk1 is a complex process that may well be regulated by other mechanisms in conjunction with the switch discussed, and that this overall regulation also depends on the organism studied.

The MF framework eliminates several parameters such as the number of phosphorylation sites 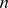 (as long as it is sufficiently large), the transition rates 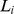 and the cooperativity coefficient 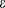. The remaining parameters, such as the shape of the activity function 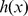, can potentially be measured in the lab using site-directed mutagenesis and activity assays. However this is still a formidable task and one that is yet to be done for most proteins involved in cell cycle regulation.

Since it is assumed in the derivation of the formula (1) that the sites are roughly independent from each other, one might think that the MF framework doesn't work for allosteric or cooperative systems. However the detailed model in Figure 2 is allosteric, and yet the model closely describes its dynamics. In simulations we find that the accuracy of the representation is increased when 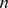 is large (e.g. 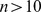) and/or the cooperativity is weak. The use of a MWC-type model for multisite phosphorylation has been pointed out in the past, see for instance [5] and the more recent [44].

Questions for future work include the following: if phosphorylation and dephosphorylation of the multisite protein 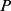 is not faster than other processes in the system, can one still approximate 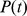 away from equilibrium? This might be possible by defining a simple differential equation for 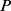 instead of the algebraic equation (1). Also, the linear dynamics used to calculate the fraction 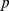 can be replaced by more complex models such as a Michaelis-Menten reaction, which may be explored in detail, including the interaction with zero-order mechanisms. This might lead to bistable behavior in the full multisite model, which raises the question of how the corresponding model reduction might be, possibly involving multivalued functions 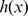.

## Methods

For convenience we include in one location all chemical reactions of the model, mass conservation laws, the definition of auxiliary variables following the multisite modeling formalism, and a self contained set of differential equations after eliminating additional variables. Recall that for multisite proteins 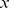 represents the fraction of active sites, 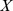 the active monomer concentration, 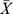 the total concentration including active and inactive forms, and 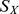 the total amount of 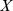 in the system including dimer and monomer forms. Also recall that Rho1 and Pkc1 are denoted by 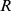, 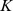, Zds1 and PP2A by 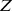, 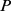, and the Rho1/Pkc1 and Zds1/PP2A dimers by 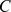, 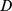, respectively.

### Model reactions



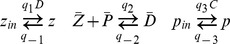


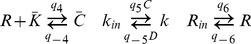


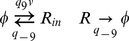



### Mass conservation laws



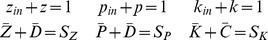



### MF framework equations



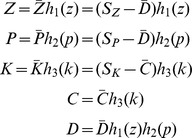



### Differential equations



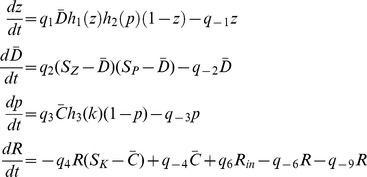


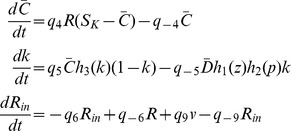



### Parameter values

Since most quantitative information about the pathway is unknown, we make educated estimates on the order of magnitude of the parameters. Since parameters are clustered in equations (2) and (3), dependence on the parameters is more limited. Protein concentrations usually range from 0.001 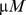 to 10 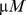 in the cell. The concentrations of total PP2A (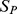), Zds1 (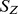) and Pkc1 (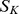) are set between 0.01 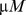 and 0.1 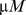 as indicated in Table 1. Define 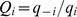 for all 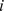. The dissociation rate 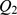 has been observed to be quite low in experiments since most Zds1 has been found bound to PP2A. We set it as 0.001 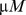, which is in the range of drugs binding to their target proteins. 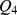 is set higher at 0.1 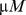. The unit-less parameters 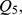 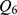 are set to 0.1 and 1 respectively, indicating the steady state ratio of inactive to active substrate when the two antagonistic enzymes are in similar concentration. The rates 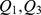 are set to 0.01 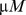, indicating that when e.g. 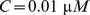 there are equal amounts of active and inactive substrate 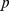 at steady state. 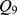 is set at 0.0002 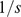.

**Table 1:** Parameter values in the cell size checkpoint model.

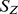	0.1 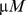	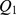	0.01 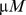	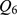	1	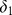	100
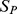	0.1 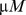	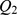	0.001 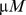	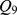	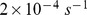	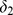	10
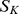	0.01 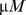	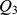	0.01 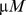	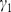	10	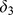	1000
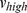	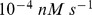	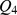	0.1 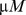	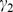	5		
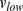	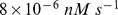	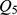	0.1	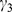	5		
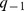	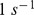	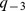	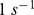	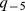	10 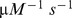	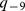	0.005 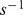
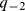	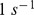	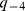	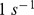	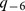	1 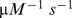		

The model parameters used in the simulation of the checkpoint pathway in Figures 3,4 are detailed in this table, including total protein concentrations, binding and unbinding rates, linear reaction rates, rates of vesicle flow and the parameters for the activation functions 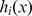. We use the notation 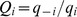. See the Methods section for an estimation of their values.

Very little is known about the values of the individual rates 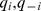. Fortunately as it is shown in the analysis in Text S1, most of the dynamic rate constants appear only in the form 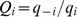, instead of individually. These steady state ratios are generally easier to estimate experimentally than the individual parameters [50]. However the actual rates 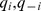 determine the transient behavior of the system and to some extent determine also its steady state values. Since a majority of the reverse rates 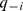 share the same units of 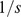, we set the values of these parameters and then find the corresponding 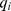 to fit the given ratio 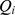. For simplicity we set 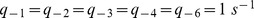 [14], [51]. We set 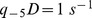 for maximal protein concentration 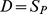, that is, 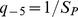. The Rho1 degradation rate 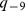 is set to 0.0001 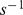; it can be further decreased in order to stabilize the Rho1 protein.

Regarding the activity functions 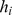, we assume that the ultrasensitive behavior of these graphs increases with the number of phosphorylation sites; see the derivation of 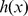 in the Results and also [5]. Since PP2A, Zds1, and Pkc1 have been found to have around 3, 5, and 8 sites respectively, we implement this with parameters that produce the graph observed in Figure 3A. See Table 1 for a list of parameter values.

The initial conditions used in the model correspond to the system in the off state. They are equal to zero for all variables, except 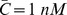 and 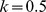.

## Supporting Information

Figure S1A: A rule-based approach for modeling multisite phosphorylation, which results in a system equivalent to MWC. Here 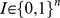 represents any phosphoform state, and 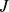 is the result of adding one site phosphorylation to 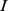. B: Timecourse simulation for the MWC model in which the kinase concentration 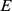 is increased linearly over time, and the full MWC model is compared with the MF approximation and a model using a single site. Here 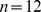, 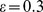,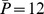 nM, 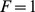 nM, 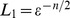, 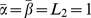 for the detailed model, and 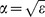, 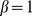, 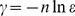, 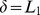 for the MF approximation.(EPS)

Figure S2For the MWC model in Figure 2B, a bootstrap parameter analysis of the corresponding MF system. Baseline parameter values are 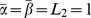, 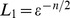. A: The parameters 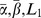 are varied over four orders of magnitude each, and the percentage error with the MF model is calculated. B: A similar analysis of the Hill exponent of the MF model.(EPS)

Text S1The supplementary material to this paper has three sections. In Section S1.1 and S1.2 we carry out a mathematical analysis of the downstream and upstream subsystems of the checkpoint model, respectively. Each analysis results in the reduction of the system at steady state to a single equation. In Section S1.3 we derive the MWC model using a system of equations involving all 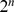 protein phosphoforms and a rule-based system of reactions.(PDF)

## References

[1] Meek DW (1998) Multisite phosphorylation and the integration of stress signals at p53. Cell Signal 10: 159–166. 9607138 10.1016/s0898-6568(97)00119-8

[2] Verma R, Annan RS, Huddleston MJ, Carr SA, Reynard G (1997) Phosphorylation of Sic1p by G1 Cdk required for its degradation and entry into S phase. Science 278: 455–460. 9334303 10.1126/science.278.5337.455

[3] Alberts B, Johnson A, Lewis J, Raff M, Roberts K, et al.. (2007) Molecular Biology of the Cell: Garland Science.

[4] Harvey S, Enciso G, Dephoure N, Gygi S, Gunawardena J, et al (2011) A phosphatase threshold sets the level of Cdk1 activity in early mitosis in budding yeast. Mol Biol of the Cell 22: 3595–3608. 10.1091/mbc.E11-04-0340 PMC3183015 21849476

[5] Serber Z, Ferrell J (2007) Tuning bulk electrostatics to regulate protein function. Cell 128: 441–444. 17289565 10.1016/j.cell.2007.01.018

[6] Iakoucheva L, Radivojac P, Brown C, O'Connor T, Sikes J, et al (2004) The importance of intrinsic disorder for protein phosphorylation. Nucleic Acid Res 32: 1037–1049. 14960716 10.1093/nar/gkh253 PMC373391

[7] Strogatz S (1994) Nonlinear Dynamics and Chaos: With Applications to Physics, Biology, Chemistry, and Engineering: Perseus Book Publishing.

[8] Angeli D, Sontag ED (2004) Multi-stability in monotone input/output systems. Systems Control Lett 51: 185–202.

[9] Lenz P, Swain P (2006) An entropic mechanism to generate highly cooperative and specific binding from protein phosphorylations. Curr Biol 16: 2150–2155. 17084700 10.1016/j.cub.2006.09.013

[10] Wang L, Nie Q, Enciso G (2010) Nonessential sites improve phosphorylation switch. Biophys J 99: 41–43. 10.1016/j.bpj.2010.07.030 PMC2941022 20858409

[11] Gunawardena J (2005) Multisite protein phosphorylation makes a good threshold but can be a poor switch. Proc Natl Acad Sci USA 102: 14617–14622. 16195377 10.1073/pnas.0507322102 PMC1253599

[12] Liu X, Bardwell L, Nie Q (2010) A combination of multisite phosphorylation and substrate sequestration produces switch-like responses. Biophys J 98: 1396–1407. 20409458 10.1016/j.bpj.2009.12.4307 PMC2856190

[13] Levchenko A (2003) Allovalency: a case of molecular entanglement. Curr Biol 13: R876–R878. 14614843 10.1016/j.cub.2003.10.049

[14] Chan C, Liu X, Wang L, Bardwell L, Nie Q, et al (2012) Protein scaffolds can enhance the bistability of multisite phosphorylation systems. PLoS Comp Biol 8: 1–9. 10.1371/journal.pcbi.1002551 PMC3380838 22737061

[15] Thomson M, Gunawardena J (2009) Unlimited multistability in multisite phosphorylation systems. Nature 460: 274–277. 19536158 10.1038/nature08102 PMC2859978

[16] Keener J, Sneyd J (2008) Mathematical Physiology I: Cellular Physiology: Springer.

[17] Murray JD (2002) Mathematical Biology I: An Introduction: Springer.

[18] Monod J, Wyman J, Changeux J-P (1965) On the nature of allosteric transitions: a plausible model. J Mol Biol 12: 88–118. 14343300 10.1016/s0022-2836(65)80285-6

[19] Sneppen K, Micheelsen MA, Dodd IB (2008) Ultrasensitive gene regulation by positive feedback loops in nucleosome modification. Mol Syst Biol 182: 4. 10.1038/msb.2008.21 PMC2387233 18414483

[20] Sourjik V (2004) Functional interactions between receptors in bacterial chemotaxis. Nature 428: 437–441. 15042093 10.1038/nature02406

[21] Jorgensen P, Tyers M (2004) How cells coordinate growth and division. Curr Biol 14: R1014–1027. 15589139 10.1016/j.cub.2004.11.027

[22] Turner JJ, Ewald JC, Skotheim JM (2012) Cell size control in yeast. Curr Biol 22 (9) R350–9. 22575477 10.1016/j.cub.2012.02.041 PMC3350643

[23] Murray A, Hunt T (1994) The Cell Cycle, An Introduction: Oxford University Press.

[24] Lew DJ, Reed SI (1993) Morphogenesis in the yeast cell cycle: regulation by Cdc28 and cyclins. J Cell Biol 120: 1305–1320. 8449978 10.1083/jcb.120.6.1305 PMC2119756

[25] Anastasia SD, Nguyen DL, Thai V, Meloy M, MacDonough T, et al (2012) A link between mitotic entry and membrane growth suggests a novel model for size control. J Cell Biol 197: 89–104. 22451696 10.1083/jcb.201108108 PMC3317797

[26] Tyson J, Novak B (2012) Irreversible transitions, bistability and checkpoint controls in the eukaryotic cycle: A systems-level understanding. In: Walhout AJM, Vidal M, Dekker J, editors. Handbook of Systems Biology. San Diego: Elsevier.

[27] Ryerson S, Enciso GA (2013) Ultrasensitive behavior of independent multisite systems. J Math Biol 2013 Sep 18. [Epub ahead of print]. 10.1007/s00285-013-0727-x 24046085

[28] Enciso GA (2013) Multisite mechanisms for ultrasensitivity in signal transduction. In Poetsche C, Kloeden P, editors. Nonautonomous and Random Dynamical Systems in Life Sciences. Springer Verlag.

[29] Strickfaden SC, Winters MJ, Ben-Ari G, Lamson RE, Tyers M, et al (2007) A mechanism for cell-cycle regulation of MAP kinase signaling in a yeast differentiation pathway. Cell 128: 519–531. 17289571 10.1016/j.cell.2006.12.032 PMC1847584

[30] Lu LX, Domingo-Sananes MR, Huzarska M, Novak B, Gould KL (2012) Multisite phosphoregulation of Cdc25 activity refines the mitotic entrance and exit switches. Proc Natl Acad Sci USA 109: 9899–9904. 22665807 10.1073/pnas.1201366109 PMC3382524

[31] Rivlin T (2003) An Introduction to the Approximation of Functions, Chapter I Mineola, NY: Dover Publications.

[32] Kapuy O, Barik D, Sananes MRD, Tyson JJ, Novak B (2009) Bistability by multiple phosphorylation of regulatory proteins. Prog Biophys Molec Biol 100: 47–56. 19523976 10.1016/j.pbiomolbio.2009.06.004 PMC2784190

[33] Goldbeter A, Koshland D (1981) An amplified sensitivity arising from covalent modication in biological systems. Proc Natl Acad Sci USA 78: 6840–6844. 6947258 10.1073/pnas.78.11.6840 PMC349147

[34] Hlavacek WS, Faeder JR, Blinov ML, Posner RG, Hucka M, et al (2006) Rules for modeling signal-transduction systems. Science Signaling 344: 1–18. 10.1126/stke.3442006re6 16849649

[35] Abe M, Qadota H, Hirata A, Ohya Y (2003) Lack of GTP-bound Rho1p in secretory vesicles of Saccharomyces cerevisiae. J Cell Biol 162: 85–97. 12847085 10.1083/jcb.200301022 PMC2172714

[36] Kamada Y, Qadota H, Python CP, Anraku Y, Ohya Y (1996) Activation of yeast protein kinase C by Rho1 GTPase. J Biol Chem 271: 9193–6. 8621575 10.1074/jbc.271.16.9193

[37] Pal G, Paraz MT, Kellogg DR (2008) Regulation of Mih1/Cdc25 by protein phosphatase 2A and casein kinase 1. J Cell Biol 180: 931–945. 18316413 10.1083/jcb.200711014 PMC2265403

[38] Wicky S, Tjandra H, Schieltz D, Yates J, Kellogg DR (2011) The Zds proteins control entry into mitosis and target protein phosphatase 2A to the Cdc25 phosphatase. Mol Biol of the Cell 22: 20–32. 10.1091/mbc.E10-06-0487 PMC3016974 21119008

[39] Edelstein-Keshet L (2005) Mathematical Models in Biology: Society for Industrial and Applied Mathematics.

[40] Elledge SJ (196) Cell cycle checkpoints: preventing an identity crisis. Science 274: 1664–1672. 8939848 10.1126/science.274.5293.1664

[41] Goldbeter A, Koshland D (1984) Ultrasensitivity in biochemical systems controlled by covalent modication: interplay between zero-order and multistep effects. J Biol Chem 259: 441–447. 6501300

[42] Toomik R, Ek P (1997) A potent and highly selective peptide substrate for protein kinase C assay. Biochem J 322: 455–460. 9065763 10.1042/bj3220455 PMC1218212

[43] Yu UY, Ahn JH (2010) Phosphorylation on the PPP2R5D B regulatory subunit modulates the biochemical properties of protein phosphatase 2A. BMB Rep 43: 263–267. 20423611 10.5483/bmbrep.2010.43.4.263

[44] Martens B, Swain P (2013) Ultrasensitivity in phosphorylation-dephosphorylation cycles with little substrate. PLoS Comp Biol 9: 1–12. 10.1371/journal.pcbi.1003175 PMC3738489 23950701

[45] Barik D, Baumann WT, Paul MR, Novak B, Tyson J (2010) A model of yeast cell-cycle regulation based on multisite phosphorylation. Mol Syst Biol 6: 1–18. 10.1038/msb.2010.55 PMC2947364 20739927

[46] Kim SY, Ferrell JE (2007) Substrate competition as a source of ultrasensitivity in the inactivation of wee1. Cell 128: 1133–1145. 17382882 10.1016/j.cell.2007.01.039

[47] Salazar C, Hofer T (2007) Versatile regulation of multisite protein phosphorylation by the order of phosphate processing and protein-protein interactions. FEBS J 274: 1046–1061. 17257173 10.1111/j.1742-4658.2007.05653.x

[48] Chen KC, Calzone L, Csikasz-Nagy A, Cross FR, Novak B, et al (2004) Integrative analysis of cell cycle control in budding yeast. Mol Biol of the Cell 15: 3841–3862. 10.1091/mbc.E03-11-0794 PMC491841 15169868

[49] Yang L, MacLellan WR, Han Z, Weiss JN, Qu Z (2004) Multisite phosphorylation and network dynamics of cyclin-dependent kinase signaling in the eukaryotic cell cycle. Biophys J 86: 3432–3443. 15189845 10.1529/biophysj.103.036558 PMC1304250

[50] Cornish-Bowden A (1979) Fundamentals of Enzyme Kinetics: Butterworth & Co.

[51] Alon U (2007) An Introduction to Systems Biology: Chapman and Hall/CRC.

