# Supplementary material for: Compact Modeling of Allosteric Multisite Proteins: Application to a Cell Size Checkpoint
Source: PLoS Comput Biol. 2014 Feb 6;10(2):e1003443. doi: 10.1371/journal.pcbi.1003443 (PMC3916233; doi:10.1371/journal.pcbi.1003443)
Supplement: Text S1 — The supplementary material to this paper has three sections. In Section S1.1 and S1.2 we carry out a mathematical analysis of the downstream and upstream subsystems of the checkpoint model, respectively. Each analysis results in the reduction of the system at steady state to a single equation. In Section S1.3 we derive the MWC model using a system of equations involving all protein phosphoforms and a rule-based system of reactions. (PDF) [file pcbi.1003443.s003.pdf]

"Compact Modeling of Allosteric Multisite Proteins: Application to a Cell Size Checkpoint", by G.A. Enciso, A. Vargas, and D.R. Kellogg.

# Text S1: Supplementary Information

The additional material to the main text is separated into three sections. In Section S1.1, we carry out a detailed analysis of the Zds1 – PP2A system, also referred to as the downstream system, and we derive equation (2) to write any steady state of the system in terms of its input, the active Rho1/Pkc1 concentration $C$. Section S1.2 has a similar objective with the upstream system involving Rho1 – Pkc1, and we derive formula (3) that solves the steady states of the system given two inputs: the flux $v$ and the active PP2A/Zds1 concentration $D$.

In Section S1.3 we consider a model based on a systematic list of chemical reactions, which might otherwise have been used to define a network using rule-based software. The reactions involve activation and deactivation of any protein in a given phosphorylation state, as well as phosphorylation and dephosphorylation at any site available for that reaction. We show that in that particular case one can exactly reduce the system to a smaller number of variables, forming the MWC model. In this way, the MF model is not only the reduction of model MWC with $2(n+1)$ variables, but also the reduction of a rule-based model with $2^{n+1}$ variables.

## S1.1: Analysis of the Zds1 – PP2A interaction subsystem

Recall that $Z,P$ are the active monomer concentrations of Zds1, PP2A respectively, $\bar{Z},\bar{P}$ the total monomer concentrations, and $p,z$ the fraction of modified sites. Assume the reactions

$$\bar{Z}+\bar{P}\underset{q_{-2}}{\overset{q_2}{\rightleftarrows}}\bar{D},\quad z_{in}\underset{q_{-1}}{\overset{q_1 D}{\rightleftarrows}}z,\quad p_{in}\underset{q_{-3}}{\overset{q_3 C}{\rightleftarrows}}p.$$

From the MF framework, estimate

$$Z=\bar{Z}h_1(z),\quad P=\bar{P}h_2(p),$$

where $h_i(x)=e^{\gamma_i x}/(\delta_i+e^{\gamma_i x})$. The Zds1/PP2A dimer is assumed to be active exactly when both monomers are active, so if $D$ is the active form concentration

$$\frac{D}{\bar{D}}=\frac{Z}{\bar{Z}}\frac{P}{\bar{P}}=h_1(z)h_2(p),$$

that is,

$$D = \bar{D} h_1(z) h_2(p).$$

The differential equations for this system are:

$$\begin{aligned}
\frac{d\bar{Z}}{dt} &= -q_2 \bar{Z}\bar{P} + q_{-2}\bar{D} \\
\frac{d\bar{P}}{dt} &= -q_2 \bar{Z}\bar{P} + q_{-2}\bar{D} \\
\frac{d\bar{D}}{dt} &= q_2 \bar{Z}\bar{P} - q_{-2}\bar{D} \\
\frac{dz}{dt} &= q_1 D z_{in} - q_{-1} z \\
\frac{dp}{dt} &= q_3 C p_{in} - q_{-3} p.
\end{aligned}$$

Notice that the dynamics of $z$ depends on $D$, the active dimer. The dynamics of $p$ depends on the concentration of the protein $C$, the active Rho1/Pkc1 dimer, which is treated for now as a given constant. One can eliminate the variables $z_{in}, p_{in}$ and replace them with $z, p$ using the conservation equations $z_{in} + z = 1$, $p_{in} + p = 1$. Similarly, one can use the mass conservation of Zds1 and PP2A, $\bar{Z} + \bar{D} = S_Z$, $\bar{P} + \bar{D} = S_P$ to eliminate $\bar{Z}$ and $\bar{P}$ from the system. Here $S_Z, S_P$ are the total amounts of Zds1 and PP2A including monomer and dimer forms. Finally, the active dimer $D$ can be written in terms of the other variables as described above. The new equations are

$$\begin{aligned}
\frac{d\bar{D}}{dt} &= q_2 (S_Z - \bar{D})(S_P - \bar{D}) - q_{-2}\bar{D} \\
\frac{dz}{dt} &= q_1 \bar{D} h_1(z) h_2(p)(1 - z) - q_{-1} z \\
\frac{dp}{dt} &= q_3 C(1 - p) - q_{-3} p.
\end{aligned}$$

Now, over time the first and third equation of this system converge towards a constant independent of the other variables. Solving for $p$ at steady state leads to $p = C/(C + q_{-3}/q_3)$. The quadratic equation for $\bar{D}$ at steady state has two solutions, however one of them is larger than $S_Z, S_P$ and can be discarded. Thus over time $\bar{D}$ converges towards a steady state value $\bar{D}_0$ independent of $C$.

Putting all together, the system is reduced to a single differential equation for $z$. As stated in the main text, the solutions of the system converge towards the steady state whenever $z$

does, and one can obtain bistability depending on parameter values. The differential equation for $z$ can be rewritten as

$$\frac{dz}{dt} = h_2\left(\frac{C}{C + q_{-3}/q_3}\right) h_1(z)(1-z) - \Gamma_1 z,$$

where $\Gamma_1 = \frac{q_{-1}}{q_1} / \bar{D}_0$. Both terms in the right hand side are described in Figure 3B as separate functions, and the steady states correspond to the intersection of the two graphs. By comparing both functions on the left and right hand sides of each steady state, one can determine that the first and third steady states are stable, and that the second steady state is unstable. This is a general property of the system whenever there are three steady states, since the sigmoidal shape of $h_1(z)$ is preserved under other parameter values, and therefore when multiplied by the constant $h_2(p)$ and the function $(1-z)$, the qualitative shape of the graph is preserved. Therefore one can conclude that the Zds1/PP2A interaction system is bistable whenever there are three steady states.

The canonical output of the core model is the active PP2A/Zds1 dimer concentration $D$. This value can be calculated from the equation above, but more easily from the steady state values of $z$ using the equation $0 = z' = q_1 D(1-z) - q_{-1} z$, that is $D = \frac{q_{-1}}{q_1} z/(1-z)$. This is the equation used to plot the graphs of $C$ versus $D$ in Figure 3C.

## S1.2: Analysis of the Rho1 – Pkc1 interaction subsystem

This system involves the proteins Rho1, Pkc1, denoted $R, K$ respectively in their active forms. Total Rho1 and Pkc1 monomer concentration, regardless of whether active or inactive, are $\bar{R}, \bar{K}$, while $k$ denotes the fraction of modified Pkc1 sites. The given chemical reactions are

$$R_{in} \underset{q_{-6}}{\overset{q_6}{\rightleftarrows}} R, \quad \phi \underset{q_{-9}}{\overset{q_9 v}{\rightleftarrows}} R_{in}, \quad R \underset{q_{-9}}{\rightarrow} \phi, \quad k_{in} \underset{q_{-5}D}{\overset{q_5 C}{\rightleftarrows}} k, \quad R + \bar{K} \underset{q_{-4}}{\overset{q_4}{\rightleftarrows}} \bar{C}.$$

The MF approximation is used to calculate

$$K = \bar{K} h_3(k), \quad C = \bar{C} h_3(k).$$

A few conservation of mass equations hold:

$$k_{in} + k = 1, \ \bar{K} + \bar{C} = S_K.$$

Using mass action kinetics one can derive a system of differential equations describing the behavior of this system. In the following, the variables $k_{in}$ and $\bar{K}$ are eliminated and replaced by $1-k$ and $S_K - \bar{C}$, respectively:

$$\begin{aligned}
\frac{dR_{in}}{dt} &= -q_6 R_{in} - q_{-9} R_{in} + q_{-6} R + q_9 v \\
\frac{dR}{dt} &= q_6 R_{in} - q_{-9} R - q_{-6} R - q_4 R(S_K - \bar{C}) + q_{-4}\bar{C} \\
\frac{dk}{dt} &= q_5 C(1-k) - q_{-5} Dk \\
\frac{d\bar{C}}{dt} &= q_4 R(S_K - \bar{C}) - q_{-4}\bar{C}
\end{aligned}$$

A steady state analysis of this system is now carried out. Even though the system contains multiple variables, it is still possible to reduce all equations to a single 1D equation at steady state. After setting all left hand sides to zero, notice that the equation for $k$ only depends on $C$ (and the fixed input parameter $D$). One can solve it to find

$$k = \frac{C}{C + Q_5 D},$$

where we introduce the notation $Q_i = q_{-i} / q_i$ for every $i$.

Next, the steady state equations for $R_{in}$ and $R$ depend linearly on the variables $R_{in}, R$. They can be solved together using elementary methods for linear equations, where the different `coefficients' may contain the variable $\bar{C}$. In particular

$$R = \frac{q_6 q_{-4}\bar{C} + q_{-9} q_{-4}\bar{C} + q_6 q_9 v}{q_4 q_6 (S_K - \bar{C}) + q_4 q_{-9}(S_K - \bar{C}) + q_{-6} q_{-9} + q_{-9}(q_6 + q_{-9})}.$$

The determinant of the linear system is the denominator of the equation, and it is larger than zero ($S_K - \bar{C} = \bar{K} \geq 0$) so that the solution is always unique. For convenience, call $Y$ the denominator of this expression. Replacing into the equation for $\bar{C}$, we have

$$Y\frac{d\bar{C}}{dt} = q_4 (S_K - \bar{C})[q_6 q_{-4}\bar{C} + q_{-9} q_{-4}\bar{C} + q_6 q_9 v] - Y q_{-4}\bar{C}.$$

By multiplying out on the right hand side, the first two terms of each summand cancel out, so that

$$Y\frac{d\bar{C}}{dt} = q_4q_6q_9(S_K - \bar{C})v \; - \; q_{-4}q_{-6}q_{-9}\bar{C} + q_{-4}q_{-9}\bar{C}(q_6 + q_{-9}).$$

Therefore

$$\frac{Y}{q_4q_6q_9}\frac{d\bar{C}}{dt} = (S_K - \bar{C})v \; - \; \Gamma_2\bar{C},$$

where

$$\Gamma_2 = Q_4Q_6Q_9 + Q_4Q_9\left(1 + \frac{q_{-9}}{q_6}\right).$$

By multiplying on both sides by $h_3(k)$ and using the identity $C = \bar{C}h_3(k)$, one obtains

$$\frac{h_3(k)Y}{q_4q_6q_9}\frac{d\bar{C}}{dt} = (S_K h_3(k) - C)v \; - \; \Gamma_2 C.$$

Finally, replacing $k$ by its expression in terms of $C$, at steady state

$$\Gamma_2 C = v\left(S_K h_3\left(\frac{C}{C + Q_5 D}\right) - C\right).$$

In order to determine the stability of the steady states, notice that $h_3(k)Y / q_4q_6q_9 > 0$, even though this expression might depend on $k$ and $\bar{C}$. By comparing the sign of the two summands in the right hand side of the expression for $d\bar{C}/dt$, one can determine when the steady states are stable or unstable. In the example in Figure 3D, once again one can see that out of the three steady states, two are stable and one is unstable.

## S1.3: Rule-Based Derivation of MWC

We begin by introducing in detail the assumptions that lead to the MWC model in the context of multisite phosphorylation. Recall that a substrate $P$ is assumed to be phosphorylated nonsequentially by a kinase $E$, that the phosphorylation is cooperative, and that the number of sites is large enough that each individual phosphorylation has a small effect on substrate activation. The substrate may be either active or inactive at any given time, due to structural transitions or the binding of the substrate to another molecule.

Based on these assumptions, we start with a basic model containing a large number of variables and reduce it to the MWC model. This framework also relates our modeling framework to the approach known as rule-based modeling, where a series of chemical reactions is defined using a

streamlined algorithm, and high-powered computing is used to handle the resulting large number of variables.

Suppose that $J \in \{0,1\}^n$ is any vector of $n$ zeros and ones indicating the phosphorylation state of the substrate, also known as its phosphoform. $A_J$ and $I_J$ indicate the concentration of the active and inactive protein phosphoforms in state $J$, respectively. For any $J,K \in \{0,1\}^n$, we say that $J \prec K$ if $J_i \le K_i$ for every $i$ and $|K|=|J|+1$, that is the $K$-phosphoform has exactly one more phosphorylation than the $J$-phosphoform.

Define the chemical reactions displayed in Figure S1A, where $J$ is any fixed index and $K$ is such that $K \succ J$. That is, every $A_J$ can transition into $I_J$ and back, and $A_J$ can also become phosphorylated at any of its remaining locations at a rate proportional to $E$. Every additional phosphorylation makes the transition of active to inactive substrate slightly more difficult, since the transition rate is multiplied by $\varepsilon^{|J|}$, where $\varepsilon < 1$. Since each phosphorylation is assumed to have a small effect, we can assume that $\varepsilon$ is close to 1.

Often there are so many reactions in such an approach that it is not possible to carry out a mathematical analysis, but we can do it in this case. Putting together all reactions, we have for every index $J$:

$$\frac{dA_J}{dt} = -\varepsilon^{|J|} L_1 A_J + L_2 I_J - \sum_{K \succ J} \bar{\alpha} E A_J + \sum_{K \succ J} \bar{\beta} F A_K + \sum_{K \prec J} \bar{\alpha} E A_K - \sum_{K \prec J} \bar{\beta} F A_J$$

$$\frac{dI_J}{dt} = \varepsilon^{|J|} L_1 A_J - L_2 I_J - \sum_{K \succ J} \bar{\alpha} \varepsilon E I_J + \sum_{K \succ J} \bar{\beta} F I_K + \sum_{K \prec J} \bar{\alpha} \varepsilon E I_K - \sum_{K \prec J} \bar{\beta} F I_J$$

The idea is to cluster together many variables $A_J$ by carrying out a change of variables. Define for $i = 0, \ldots n$,

$$A_i = \sum_{|J|=i} A_J, \quad I_i = \sum_{|J|=i} I_J.$$

Then

$$\frac{dA_i}{dt} = \sum_{|J|=i} \frac{dA_J}{dt} = -\varepsilon^{|J|} L_1 A_i + L_2 I_i - \sum_{|J|=i} \sum_{K \succ J} \bar{\alpha} E A_J + \sum_{|J|=i} \sum_{K \succ J} \bar{\beta} F A_K$$
$$+ \sum_{|J|=i} \sum_{K \prec J} \bar{\alpha} E A_K - \sum_{|J|=i} \sum_{K \prec J} \bar{\beta} F A_J$$

Therefore

$$\frac{dA_i}{dt} = -\varepsilon^i L_1 A_i + L_2 I_i - \sum_{|J|=i} (n-i) \bar{\alpha} E A_J + \sum_{|K|=i+1} \sum_{J \prec K} \bar{\beta} F A_K + \sum_{|K|=i-1} (n-i+1) \bar{\alpha} E A_K - \sum_{|J|=i} i \bar{\beta} F A_J$$

That is, for $i = 1 \ldots n-1$,

$$\frac{dA_i}{dt} = -\varepsilon^i L_1 A_i + L_2 I_i - (n-i)\bar{\alpha} E A_i + (i+1)\bar{\beta} F A_{i+1} + (n-i+1)\bar{\alpha} E A_{i-1} - i\bar{\beta} F A_i.$$

The rate equations for $A_0$, $A_n$, and $I_i$ for $i = 0 \ldots n$ can be calculated in the same way. The full system has the following equations:

$$\begin{aligned}
\frac{dA_0}{dt} &= -L_1 A_0 + L_2 I_0 - n\bar{\alpha} E A_0 + \bar{\beta} F A_1 \\
\frac{dA_i}{dt} &= -\varepsilon^i L_1 A_i + L_2 I_i - (n-i)\bar{\alpha} E A_i + (i+1)\bar{\beta} F A_{i+1} + (n-i+1)\bar{\alpha} E A_{i-1} - i\bar{\beta} F A_i, \ i = 1 \ldots n-1 \\
\frac{dA_n}{dt} &= -\varepsilon^n L_1 A_n + L_2 I_n + \bar{\alpha} E A_{n-1} - n\bar{\beta} F A_n \\
\frac{dI_0}{dt} &= L_1 A_0 - L_2 I_0 - n\bar{\alpha}\varepsilon E I_0 + \bar{\beta} F I_1 \\
\frac{dI_i}{dt} &= \varepsilon^i L_1 A_i - L_2 I_i - (n-i)\bar{\alpha}\varepsilon E I_i + (i+1)\bar{\beta} F I_{i+1} + (n-i+1)\bar{\alpha}\varepsilon E I_{i-1} - i\bar{\beta} F I_i, \ i = 1 \ldots n-1 \\
\frac{dI_n}{dt} &= \varepsilon^n L_1 A_n - L_2 I_n + \bar{\alpha}\varepsilon E I_{n-1} - n\bar{\beta} F I_n.
\end{aligned}$$

This is exactly the system of equations for $A_i, I_i$ in the diagram shown in Figure 2B. In this way, MWC is a sequential shorthand model for a nonsequential system with $2 \times 2^n$ variables.

The same framework can be used if the sites are modified through any covalent enzymatic reaction such as acetylation or methylation. They could also be non-covalent ligand binding sites, such as a receptor complex, with a background ligand $E$ in large enough amount that its concentration is not affected by its binding to the receptor.

Recall that to calculate the activation function $h(x)$, in the main text we assumed an approximate balance at steady state for the following reaction of activation and inactivation after $i$ phosphorylations:

$$A_i \underset{L_2}{\overset{L_1 \varepsilon^i}{\rightleftarrows}} I_i$$

After calculating the fraction of active sites with this number of phosphorylations, this shows that

$$h(x) = \frac{e^{\gamma x}}{\delta + e^{\gamma x}},$$

where $\gamma = -n \ln \varepsilon$ and $\delta = L_1 / L_2$. The balance between $A_i$ and $I_i$ is guaranteed for the parameters as defined above since the system MWC satisfies the property of detailed balance [1]. However it can also be approximately satisfied when detailed balance fails, especially when the transition rates $L_1, L_2$ are large compared with other parameters. Detailed balance is usually satisfied for systems that conserve energy such as multisite ligand binding. Systems that require energy to function, such as phosphorylation which requires ATP, are known as “dissipative” and don't necessarily satisfy this property, in which case the transition rates may play a more important role when compared with other rates in the system. In the case of the cell cycle checkpoint proteins, the standing assumption is that their activity is determined by internal transitions or by binding to a regulatory protein, which would need to be compared with e.g. the rate of the different enzymatic reactions.

## References

[1] N.G. Van Kampen, Stochastic Processes in Physics and Chemistry, Third Edition, Elsevier, Amsterdam, 2007.
